# Immune impairment and adaptive response in relation to antiretroviral therapy of HIV-infection

**DOI:** 10.1186/1758-2652-13-S4-P61

**Published:** 2010-11-08

**Authors:** GD Kaminskiy, AJ Pronin

**Affiliations:** 1 Moscow Regional Aids Centre, Schepkina 61/2 build.8, Moscow, Russian Federation

## Purpose of the study

In Moscow Regional HIV Living Cohort we have recently defined the groups with progressive HIV infection and those with temporary and permanently no progressive disease. The aim of the study was to find major features of progressive HIV infection in relation to antiretroviral treatment.

## Methods

In the study 615 progressors were compared with 1311 and 345 temporary and permanently non progressors. Additionally 208 late presenters (with CD4 counts less than 100 cells/mm^3^) were compared with the sample from the whole population of people leaving with HIV (2271 patient). In blood specimens HIV virus load (PCR m2000rt Abbott, 'RealTime HIV-1') and major subpopulation of T-lymphocytes were analyzed (flow cytometer BD FACSCount, sets ÑD3/CD4/CD8/CD45).

## Summary of results

The most distinct feature allocating groups was the response to the elevation of viral load. In the progressive group the number of CD8 cells among individuals decreased with the elevation of viral load. This was due to the T-cell depression including CD8+, CD4+ and CD3+CD4-CD8- T-lymphocytes.

**Figure 1:**
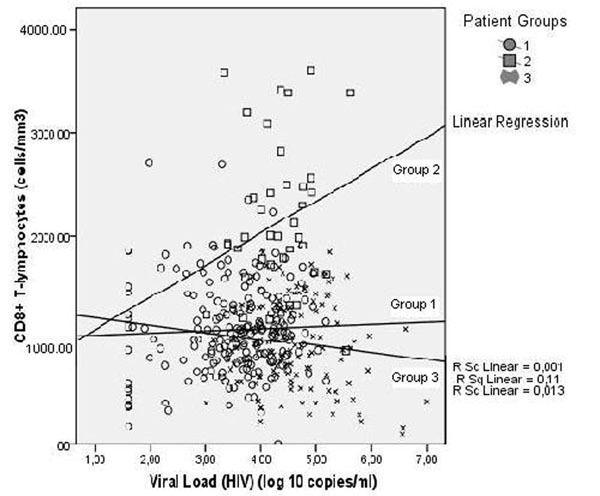


Contrary to this no progressive groups demonstrated elevation of CD8 population with the increase of viral load. The elevation was more expressed in permanently no progressive group. This resulted in preservation of CD4 T-lymphocyte subset and CD3+CD4-CD8- T-lymphocytes were significantly elevated. Figure 1.

Demonstrated "pathologic process" and "adaptive response" allows better understanding of the HAART efficacy. HAART affects the viral replication thus restoring the proportion between the cytotoxic lymphocytes and virus infected cells. This causes HIV viral load reduction. Immune reconstitution appears to be a host property which depends on the severity of the previous immune impairment.

Among the studied late presenters two groups were defined. Table 1

**Table 1:** 

	CD3+ (cells/mm^3^)	CD4+ (cells/mm^3^)	CD8+ (cells/mm^3^)	Viral Load (HIV) (log 10 copies/ml)	CD3+ CD4-CD8- (cells/mm^3^)
Late Presenters First Group	225	38	289	5,47	19

Late Presenters Second Group	552	26	380	6,09	179

HIV Living Cohort	2363	589	1195	3,80	394

Though both groups had pronounced CD4 loss and high burden of the viral load, the amount of CD8+ and CD3+CD4-CD8- depletion was different between the groups and more profound in the first group compared with the second. For the first group with severe breakage of immunity combination regiments of HAART with boosted protease inhibitors, fusion and/or integration inhibitors are recommended even in the first line of therapy to obtain better results.

## Conclusions

Depletion of different T-cell branches is the major pathological process in HIV-infection, diagnosis of the level of immune impairment is needed to prescribe appropriate treatment.
